# A Unique Transformation from Ordinary Differential Equations to Reaction Networks

**DOI:** 10.1371/journal.pone.0014284

**Published:** 2010-12-22

**Authors:** Sylvain Soliman, Monika Heiner

**Affiliations:** 1 Equipe-Projet Contraintes, INRIA Paris-Rocquencourt, BP105, Paris, France; 2 Department of Computer Science, Brandenburg University of Technology, Cottbus, Germany; Virginia Tech, United States of America

## Abstract

Many models in Systems Biology are described as a system of Ordinary Differential Equations, which allows for transient, steady-state or bifurcation analysis when kinetic information is available. Complementary structure-related qualitative analysis techniques have become increasingly popular in recent years, like qualitative model checking or pathway analysis (elementary modes, invariants, flux balance analysis, graph-based analyses, chemical organization theory, etc.). They do not rely on kinetic information but require a well-defined structure as stochastic analysis techniques equally do. In this article, we look into the structure inference problem for a model described by a system of Ordinary Differential Equations and provide conditions for the uniqueness of its solution. We describe a method to extract a structured reaction network model, represented as a bipartite multigraph, for example, a continuous Petri net (CPN), from a system of Ordinary Differential Equations (ODEs). A CPN uniquely defines an ODE, and each ODE can be transformed into a CPN. However, it is not obvious under which conditions the transformation of an ODE into a CPN is unique, that is, when a given ODE defines exactly one CPN. We provide biochemically relevant sufficient conditions under which the derived structure is unique and counterexamples showing the necessity of each condition. Our method is implemented and available; we illustrate it on some signal transduction models from the BioModels database. A prototype implementation of the method is made available to modellers at http://contraintes.inria.fr/~soliman/ode2pn.html, and the data mentioned in the “Results” section at http://contraintes.inria.fr/~soliman/ode2pn_data/. Our results yield a new recommendation for the import/export feature of tools supporting the SBML exchange format.

## Introduction

Many models in Systems Biology are described as a system of Ordinary Differential Equations (ODEs), which allows for transient and steady-state analysis (for instance using MATLAB®), or bifurcation analysis with tools like XPPAUT [1], but only when kinetic information is available.

Complementary structure-related qualitative analysis techniques have become increasingly popular in recent years, such as qualitative model checking or pathway analysis. Qualitative analysis techniques do not rely on kinetic information, but require a precisely structured model with well-identified products, reactants and catalysts (and their stoichiometry, if any) for each reaction.

**Figure 1:**
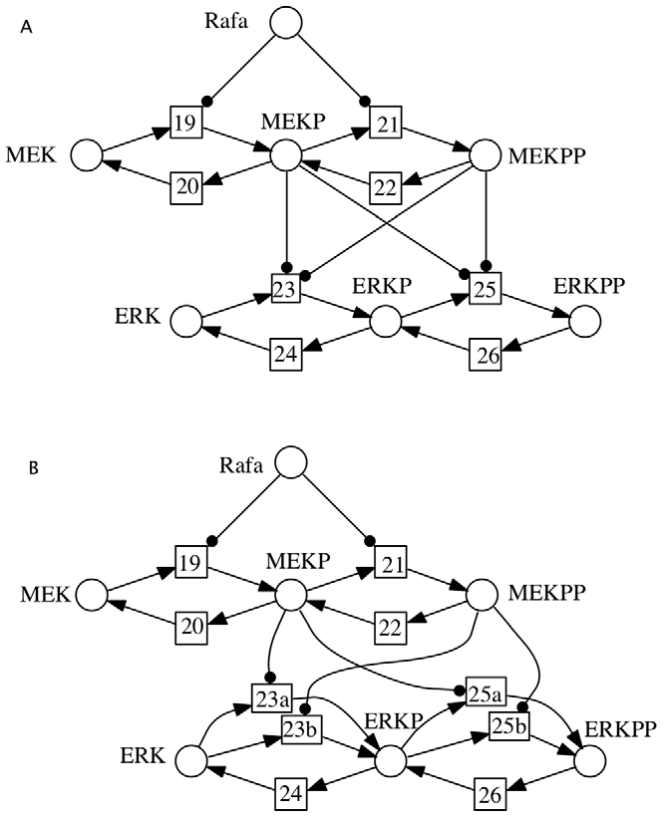
Arbitrary complex kinetics may hide essential structure. The example is an excerpt from the network model discussed in [33]. (A) Structure as suggested by the schematic representation in [33] and the list of reactions in the model's SBML format (Created by COPASI version 4.0 (Build 18) on 2006-10-24); (B) Correct structure, which is hidden in the kinetics of reactions 23 and 25. The two structures obviously differ in their discrete behaviour.

The fact that the Systems Biology Markup Language (SBML) [2] has become a standard for sharing and publishing of models has helped in making modelers clarify the structure of their models. Unfortunately, SBML does not enforce that the structure and underlying ODEs are coherent. Even if the system is specified by a list of reactions, as supported, e.g., by COPASI [3], modelers tend to specify their reaction kinetics differently when aiming at ODEs analysis. The troublemakers are reactions with complex kinetics. COPASI provides a list of predefined functions; some of them actually stand for whole building blocks. Thus, the structural interpretation of models specified in formalisms such as SBML may vary according to the source of the original model. Particularly, if the models were originally meant to be ODE-oriented, a later discrete interpretation as a qualitative or stochastic model by a naive automatic translation may produce wrong results; see Figure 1 for an introductory example demonstrating the problem.

In [4], it is elaborated that structural information hidden in kinetic laws may affect the results obtained from structural analysis, such as elementary mode analysis [5], extreme pathway analysis [6], flux balance analysis [7], chemical organization theory [8], deficiency analysis or chemical reaction network theory (CRNT) [9], [10]. This perfectly coincides with our own experience, and applies equally for place and transition invariant analysis to validate a model, see e.g. [11]–[13], or to derive automatically an hierarchically structured network representation [14].

Structural analysis may directly support ODEs-oriented dynamic analyses; e.g. [15] applies network decomposition for a modular parameter estimation approach, [16] introduces a structural persistency criterion, and transition invariants are used in [17] to identify fragile nodes and the core network responsible for the steady state behaviour, and in [18] to determine steady state solutions.

Likewise, the correct structure is mandatory when a reaction network is meant to be put into a stochastic setting, as it has been introduced in the Petri net context in the seminal paper [19], and exercised by applying various stochastic analysis techniques (standard Markovian transient and steady state analysis, analytical and simulative model checking) to a running case study in, e.g., [12], [20].

In [4], the authors present an algorithm that uncovers hidden structural information for some models already given in SBML. On the contrary, in our article we discuss conditions for unique structure inference directly from a given system of ODEs. We derive from those conditions an algorithm, that has been implemented and made public. We illustrate the necessity of our conditions and the result of the inference on some simple examples. This allows for a correct and automatic translation from ODE models to structured models suitable for qualitative or stochastic analysis, which we demonstrate on the very examples of the BioModels database [21] that were incorrectly transcribed in SBML as shown by [4].

We model a reaction network by a continuous Petri net (CPN), see [22]. We define 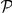, the set of places, with 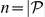, and 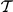, the set of transitions, with 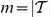. 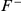 and 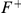 are 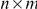 incidence matrices describing the weights of the transitions' input and output arcs, respectively. The matrix entries are denoted by 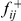 and 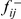, respectively.

Each transition 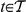 has a rate function 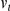 specifying the generally state-dependent continuous flow over its input and output arcs. 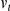 can be an arbitrary function, but its variables are restricted to the pre-places of 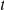 to enforce a close relation between structure and dynamic behavior. A CPN uniquely defines a system of ODEs over the variables corresponding to the places 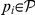:
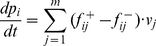
(1)


We are interested in mapping a system of ODEs onto a CPN, such that the reverse operation according to (1) gives an equivalent system (up to simple algebraic operations obviously ensuring behavioral equivalence, such as 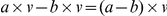). Thus, we will assume that the variables of the system of ODEs are: 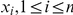, i.e., each variable is mapped in a unique way to a place 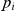 of the net, which is required by the reverse mapping.

Such mappings have already been used in the Systems Biology community, e.g. in the need for a stochastic view of models originally described by ODEs. For instance in STODE [23], which was supposed to be included in COPASI, in BlenX [24], and the Beta Workbench [25]. However, no precise algorithm is described, and program sources of implementations are not available. Most importantly, these computational platforms do not care about our main concern – the uniqueness of the revealed structure.

**Figure 2:**
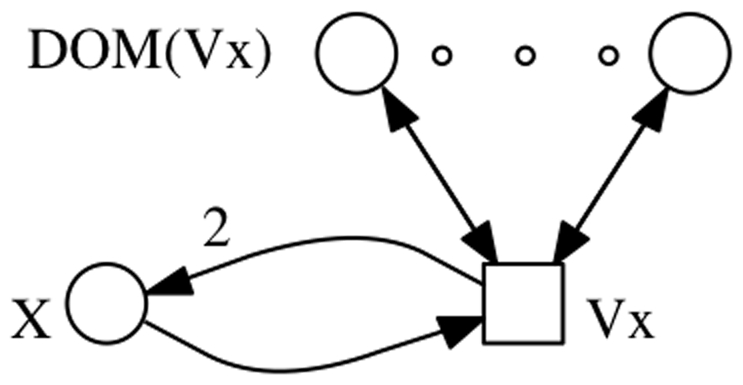
General principle to construct a CPN for an arbitrary ODEs. 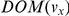
 denotes the domain of the function 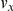.

Please note that any ODEs can be represented by a CPN simply by considering the full expression of each 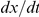, i.e. the right-hand side of the equation, as the 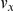 of a single transition 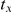 with all variables used in 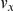 (i.e., the domain of 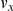) as pre-places, and exactly the same post-places (with the same arc weights), except for 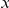 itself, which should have as weight on 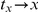 one more than the weight on 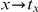; compare Figure 2. This naive translation always works and produces a net having an equal number of places and transitions, with structural information typically hidden in the generally complex kinetics 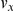. However, it is not obvious under which conditions there is exactly one CPN corresponding to a system of ODEs (even if we assume minimal arc weights), and especially whether certain biologically reasonable conditions on the CPN enforce its uniqueness. In the following we discuss ODEs conditions ensuring that there exists only one CPN; but it will almost never be the one we get by the naive translation.

## Methods

We will first present a restricted form of our results and then discuss its generalization to other types of kinetics. We will give examples where even quite simple kinetics leads to ambiguity, i.e., several nets can generate the same system of ODEs.

### Mass Action Law

In order to obtain uniqueness of the net, we will first restrict ourselves to the case where our first condition holds.

#### Condition 1

*The CPN has pure mass action law kinetics, i.e.*

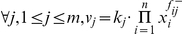



*where the parameters* 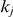 *belong to a finite alphabet* 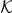 *of symbols.*

Mass action is the basis of more elaborate rates used in biological models, like Michaelis-Menten or Hill kinetics, and the use of symbolic parameters is quite standard in ODEs models since it allows the modeler to “play” with a system of ODEs in a simple and coherent way. Mass action kinetics are also necessary for some stochastic simulation methods or analysis techniques like CRNT [9].

It is obvious that for arbitrary kinetics there is little hope to find a unique CPN. Moreover the following examples show that even quite simple kinetics can lead to ambiguity, i.e., several net structures can give the same system of ODEs (see Example 1), and that there is a need for symbolic parameters to ensure uniqueness (see Example 2).

#### Example 1

*Consider the following ODEs:*

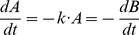
(2)


**Figure 3:**
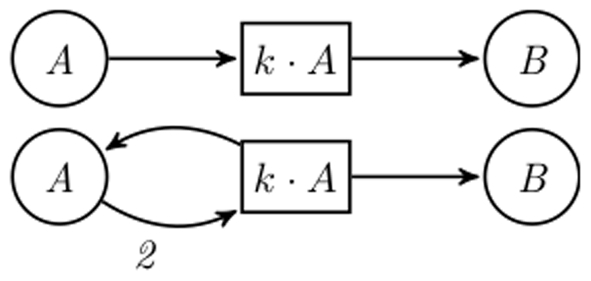
Two possible structures for Example 1. This illustrates the fact that arbitrary kinetic expressions introduce an ambiguity in the structure inference, even for very simple ODEs. The upper CPN represents the unique solution if reading equation (2) with the three established conditions.

*If one allows general kinetic expressions, even restricted such that they have the same variables as they have pre-places, one could obtain the two nets given in* *Figure 3* *.*

*Note that the second net does not respect Condition 1, since the kinetics should have been* 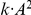.

#### Example 2

*Consider the following ODEs:*

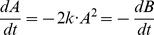
(3)


**Figure 4:**
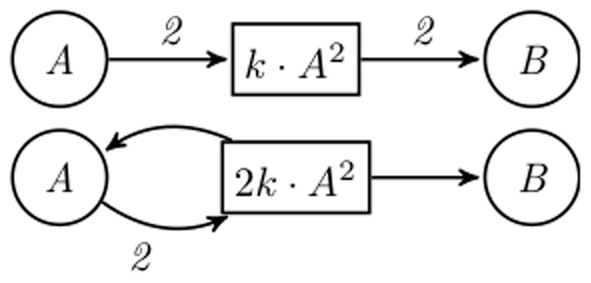
Two possible structures for Example 2. This illustrates the need for symbolic parameters in order to avoid confusion when inferring the structure.

*Symbolic parameters are required to avoid that* (3) *leads to the two nets given in* *Figure 4* *.*

We obtain the following system of ODEs by combining Condition 1 with equation (1):
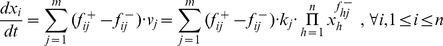



If a system of ODEs can be put in such a form, thanks to basic algebraic transformations, we will try to extract from it a CPN. Otherwise, it does obviously not correspond to any model fulfilling Condition 1.

We thus restrict our study to ODE systems of the form:
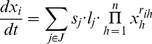
(4)


where 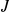 is a set of indices and for all 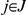 it holds 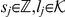, and all 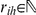; in other words, ODE systems where the right side is a polynomial over 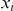, with coefficients being integer linear combinations of parameters in 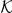.

A reaction which has exactly the same multisets of pre- and post-places, i.e., reactants and products, will only lead to null members in any ODE. Thus, we also assume:

#### Condition 2

*The CPN does not contain any void reaction, i.e.*,
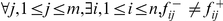



Finally, we introduce a third purely syntactic condition to ensure uniqueness of the CPN.

#### Condition 3

*In the CPN, the same parameter is never used for two different reactions with the same reactants, i.e.*,
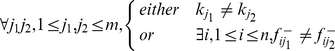



We illustrate Condition 3 by Example 3.

#### Example 3

**Figure 5:**
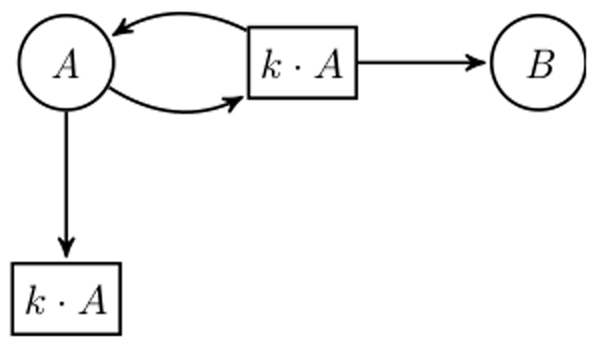
Another possible structure for the same equations as for **Figure 3**, as explained in Example 3. Even with symbolic parameters and pure mass action kinetics, if it is allowed to use the same parameter for two distinct reactions with the same reactants, one can obtain several structures for the same ODEs.

*We consider again system* (2). *Complying with Condition 1, but allowing a single parameter to be used twice for the same reactants, i.e., violating Condition 3, one could obtain the net given in* *Figure 5* *.*

Indeed, for the given system (2) and with the three introduced conditions, there are necessarily two places (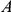 and 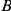), one single transition (it has kinetics 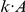), a single pre-place (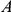 with weight 1), and a single post-place (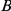 with weight 1); see the first CPN of Example 1 in Figure 3.

Before turning to our main result, we introduce two lemmata.

#### Lemma 1

*Under our three conditions, all kinetics* 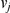 *appear at least once in the ODEs.*

#### Proof

Let us suppose that 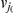 does not appear in the system. We thus have 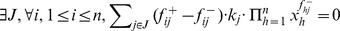 with 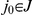.

Let us first consider the case where 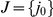, i.e., the term 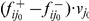 amounts to 0 for all 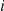. This would either violate Condition 1 if 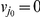, or violate Condition 2 if 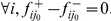


Thus there are necessarily some terms compensating for 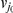 in some equations. These ODEs are precisely all the 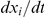 such that 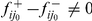.

However, since parameters are symbolic, only monomials with the same value of 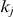 and the same degree for all 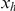 can compensate each other. But under Condition 3 there are no other 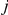 that share these features with 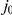.

#### Lemma 2

*Conversely, for each term* 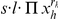 *of the ODEs, there exists a transition with parameter* 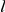 *, and pre-places* 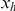 *with the corresponding arc weights* 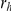.

#### Proof

The existence is obtained directly from the mapping of CPNs to ODEs according to (1). Since parameters and variables are symbolic objects, no term of that form can be created otherwise.

There is only a single such transition in any net agreeing with Condition 3. Thus, if there are several terms with the same 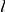 and 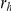: 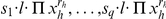, they correspond to the same transition and can be merged into one single term 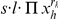 with 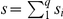.

We can now proceed to our main result.

#### Theorem 1

*For any system* 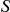 *of ODEs defining* 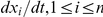 according to Conditions 1–3, there exists at most one CPN, such that the system 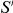 obtained from it according to (1) *is equivalent to* 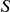,* up to basic arithmetic.*

#### Proof

We have seen that the 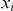 uniquely define 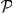. From Lemma 1 and 2 we obtain the uniqueness of the definition of 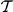 and 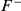.

Now, the post-places and corresponding weights are defined unambiguously by looking at 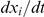 and imposing the constraint 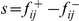, i.e., 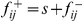 with 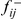 already determined to be equal to some 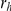 in the previous step. If the obtained 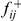 is strictly negative, there is no CPN that would produce such system under the assumed conditions.

The theorem states that there is *at most* one CPN. Indeed lots of ODEs are not amenable to (4) and thus do not comply with our first condition. However even for some systems that do comply with it there exists no model fulfilling our three conditions, as illustrated by Example 4.

#### Example 4

*An ODE system that can be put in the form of* ** *equation* (4), *but does not correspond to any CPN fulfilling our three conditions is* 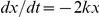.

*In this case, from the ODEs one would obtain a single place for* 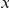,* a single transition with parameter* 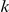,* an input weight of* 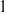, *but no possible output weight:* 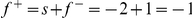.

### Beyond Mass Action Law

About 10% of the models of the BioModels database fulfill our three conditions. However it is quite common to use classical enzymatic kinetics like Michaelis-Menten or Hill type kinetics.

Actually, one can weaken Condition 1 in order to cope with Michaelian kinetics of the form: 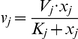 in addition to the mass action law case.

Instead of polynomials, the right members of the ODEs will then be rational fractions. But thanks to the partial fraction decomposition theorem (see for instance [26]) they can be decomposed in a unique way into a sum of a polynomial and of rational fractions, with irreducible polynomials as denominator and a numerator of strictly smaller degree.

In our case, the simple rational fractions will have degree one denominator (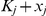) and degree zero numerator, otherwise there is no CPN corresponding to these ODEs without violating our new condition. These fractions can be easily and unequivocally transformed into the above form, the remaining polynomial will be handled as in the previous section.

## Results

We built a prototype implementation of the method outlined above – the tool ode2pn, which converts XPPAUT files into SBML (Level 2, Version 1) or APNN (one of the standard Petri net formats [27]), respectively, by applying directly the constructive proof of Theorem 1. We built upon an already existing tool, Nicotine [28], for the output of the structured model and added to it an XPPAUT parser that uses Lemma 2 to introduce a new reaction for each corresponding term in the ODEs and Theorem 1 to complete the stoichiometry matrix.

The tool rejects the conversion when no structured model fulfilling our conditions can be obtained. It is available at http://contraintes.inria.fr/~soliman/ode2pn.html.

Note that the partial fraction decomposition necessary for the Michaelian kinetics always exists, but is “practical” only with prior knowledge of the poles of the denominator's polynomials. These are the 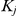 in the Michaelian case. Actually, our implementation supposes that the corresponding rational fractions are already in decomposed form.

In [4], five models from the BioModels database were identified as having been transcribed in SBML with some structural information missing: models 44, 93, 94, 143 and 151 (we adopt the convention to reduce the official model names to at most three digits). Model 44 involves Hill Kinetics and model 143 even more complex kinetic laws; so our approach cannot guarantee the uniqueness of the structure for these two cases. In the following we discuss our results for the remaining three models.

Contrary to [4], where SBML files are evaluated directly, we take the auto-generated XPP files (i.e. ODEs, generated from those SBML models), which we downloaded from the BioModels database in September 2009, and hand-curated in order to obtain exactly the ODEs as given in the original articles.

**Figure 6:**
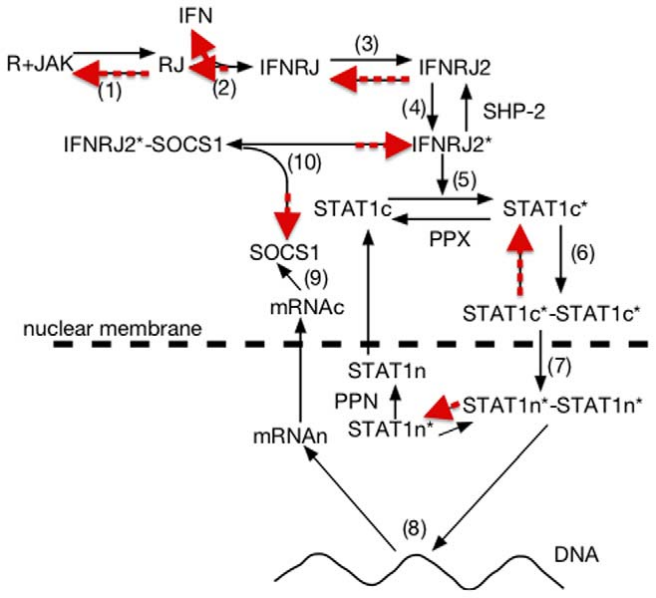
**Figure 1** of [**29**] representing a schematic view of the JAK/STAT pathway. The incorrect structure of the corresponding SBML models (93 and 94) of the BioModels database can be automatically fixed by going back to the differential equations and extracting the unique structure fulfilling our three conditions. It then correctly includes the reversibility of reactions (1), (2), (3), (6), etc. highlighted in red, and absent from the BioModels database version.

Models 93 and 94 are two models of the JAK/STAT pathway by [29]. In the original article they are described by a drawing (see Fig. 6) and a mixture of what the authors call “chemical reactions” and of ODEs (mostly for mRNAs). They are used as ODEs for simulation and were hand-transcribed to SBML for inclusion in BioModels database, but missing the “reversibility” of some reactions. We input the 34 differential equations (in each case) to our tool, with sometimes more than ten different terms in a single equation, and obtained the unique structure complying with our conditions (with the Michaelian extension) and correctly including reverse reactions when needed.

**Figure 7:**
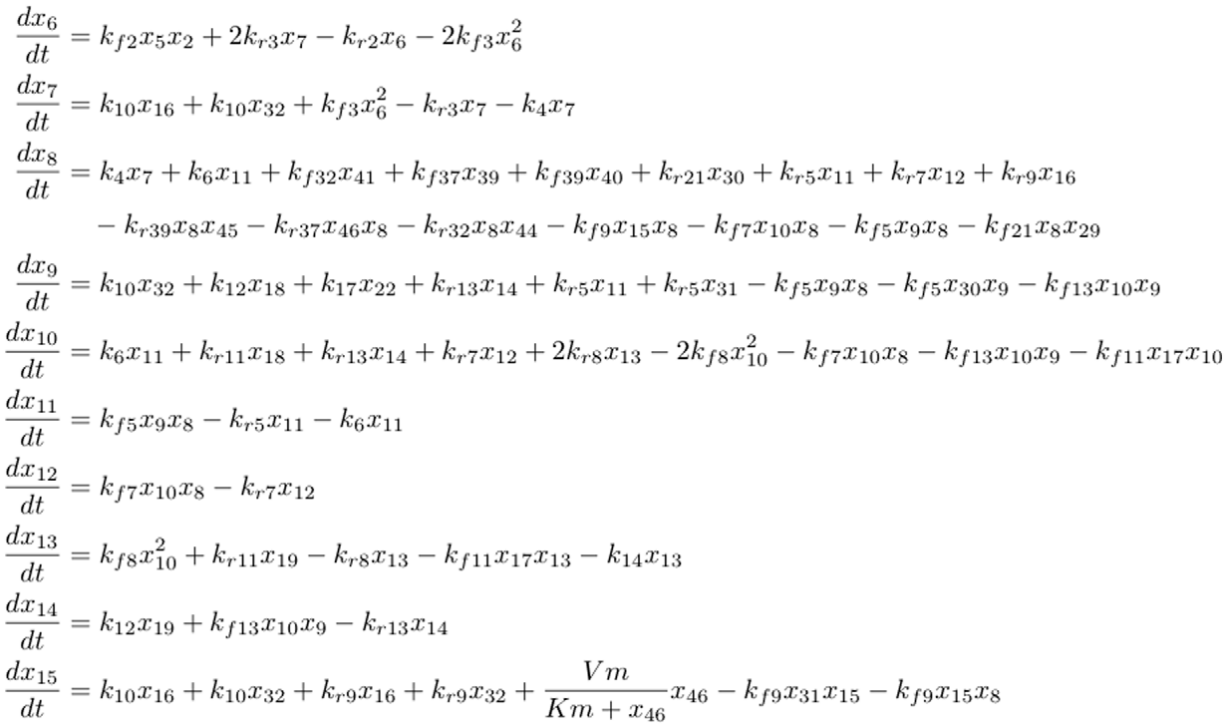
Beginning of the Appendix II of [**30**] describing the full ODE model of that article. The 68 ODEs actually allow the extraction of a unique model fulfilling the three established conditions. It not only correctly reflects the structure described in the article, but also avoids the typos introduced in the hand-written model 151 of the BioModels database; hand-typing an SBML model for that many ODEs with numerous parameters is definitely error-prone.

Model 151 is a model of the regulation of that same JAK/STAT pathway by IL-6 in hepatocytes [30]. It includes 68 differential equations (see Fig. 7 for an extract) and once again leads to a unique structure (with mass action and Michaelian kinetics). The XPPAUT.ode file (BIOMD151.ode) and the resulting structured SBML file (BIOMD151_new.xml) can be found at http://contraintes.inria.fr/~soliman/ode2pn_data/together with the biomodels version (BIOMD0000000151.xml), which actually contains more errors than found by [4], mostly concerning parameter names that are quite error-prone when hand-translated from ODEs to SBML. Note that the XPPAUT file which we provide corrects two typos from the original article, namely 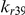 instead of 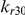 in 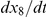 and 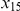 instead of 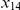 in 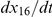. These typos still allow extraction of a unique structured model, but with obvious differences compared to that described in the article.

The converted models can be further processed by any tool complying with SBML or APNN, e.g. using Snoopy [31], which supports both formats and allows for graphical visualization of the translation results.

## Discussion

We have discussed conditions for a unique structure inference out of a given system of ODEs. For reaction networks fulfilling the given three conditions, ODEs and a structured formalization by, e.g., a CPN, are equivalent representations, which can be transformed into each other without loss of information. Note that these networks are restricted to mass action or Michaelian kinetics, which are the most widely used kinetics for biochemical systems, and prohibit empty reactions which would not have any biochemical meaning. These conditions forbid models, which were mathematically correct, but contradict reasonable biochemical expectations.

We have shown that otherwise the structure is not uniquely defined by a system of ODEs. We have given examples where violating our conditions leads to several nets having possibly different discrete, and thus stochastic behavior, but generating the same system of ODEs. These counterexamples demonstrate the necessity of each individual condition. We have given a constructive proof for the translation algorithm, which has been directly implemented, providing XPP to SBML conversion.

Our conditions are quite restrictive (only Mass-Action and Michaelian kinetics), but do cover a large part of mathematical biology models. This should allow, in the future, more and more modelers to benefit from structural analysis techniques for their systems, even if done as an afterthought. It also leads to more precise links between the different formalisms and launches a bridge betweens different communities of the Systems Biology field. In those cases where both the ODEs and a reaction diagram are given, our method allows the check if they are consistent.

Ideally, models are specified with our conditions in mind, be it as a list of reactions (as, e.g., in COPASI) or some graphical notation (e.g., continuous Petri nets). In both cases, kinetic functions should obey the three established conditions. User-friendly tools might check these conditions while doing export to SBML files to prevent misleading results by later use. Sophisticated ODE tools will have no problems in applying adequate algebraic transformations to optimize the simulation algorithms' run-time behavior. Any import of SBML files should check these conditions if aiming at structure-related qualitative or stochastic analysis techniques.

We intend to continue in trying to find uniqueness conditions for more general kinetics, and to devise heuristics for structure inference when uniqueness cannot be obtained (unwinding algebraic conservation laws coming from rapid equilibria, for instance). We also plan to make our algorithm more widely usable, for instance through a CellDesigner [32] XPP-import plugin.

## References

[1] Ermentrout B (2002). Simulating, Analyzing, and Animating Dynamical Systems: A Guide to XPPAUT for Researchers and Students.. http://www.math.pitt.edu/bard/xpp/xpp.html.

[2] Hucka M, Finney A, Sauro HM, Bolouri H, Doyle JC (2003). The systems biology markup language (SBML): A medium for representation and exchange of biochemical network models.. Bioinformatics.

[3] Hoops S, Sahle S, Gauges R, Lee C, Pahle J (2006). Copasi – a complex pathway simulator.. BioInformatics.

[4] Kaleta C, Richter S, Dittrich P (2009). Using chemical organization theory for model checking.. Bioinformatics.

[5] Schuster S, Fell DA, Dandekar T (2002). A general definition of metabolic pathways useful for systematic organization and analysis of complex metabolic networks.. Nature Biotechnology.

[6] Palsson B (2006). Systems Biology: Properties of Reconstructed Networks..

[7] Varma A, Palsson B (1994). Metabolic flux balancing: basic concepts, scientific and practical use.. Nature Biotechnology.

[8] Dittrich P, di Fenizio P (2007). Chemical organisation theory.. Bulletin of Mathematical Biology.

[9] Feinberg M, Lapidus L, Amundson NR (1977). Mathematical aspects of mass action kinetics.. Chemical Reactor Theory: A Review.

[10] Shinar G, Feinberg M (2010). Structural sources of robustness in biochemical reaction networks.. Science.

[11] Heiner M, Koch I (2004). Petri Net Based Model Validation in Systems Biology..

[12] Heiner M, Gilbert D, Donaldson R (2008). Petri nets in systems and synthetic biology..

[13] Clark A, Gilmore S, Guerriero ML, Kemper P (2010). On verifying bio-pepa models.. CMSB '10: Proceedings of the 8th International Conference on Computational Methods in Systems Biology.

[14] Heiner M, Condon A (2009). Understanding network behaviour by structured representations of transition invariants.. Algorithmic Bioprocesses; Natural Computing series.

[15] Koh G, Teong H, Clement MV, Hsu D, Thiagarajan P (2006). A Decompositional Approach to Parameter Estimation in Pathway Modeling: a Case Study of the Akt and MAPK Pathways and their Crosstalk.. Bioinformatics.

[16] Angeli D, De Leenheer P, Sontag E (2007). A Petri net approach to persistence analysis in chemical reaction networks.. Biology and Control Theory: Current Challenges; LNCI 357.

[17] Heiner M, Sriram K (2010). Structural analysis to determine the core of hypoxia response network.. PLoS ONE.

[18] Nabli F, Soliman S (2010). Steady-state solution of biochemical systems, beyond s-systems via t-invariants.. CMSB '10: Proceedings of the 8th International Conference on Computational Methods in Systems Biology.

[19] Goss PJE, Peccoud J (1998). Quantitative modeling of stochastic systems in molecular biology by using stochastic Petri nets.. Proc Natl Acad Sci USA.

[20] Heiner M, Donaldson R, Gilbert D, Iyengar MS (2010). Petri Nets for Systems Biology,. Symbolic Systems Biology: Theory and Methods.

[21] le Novère N, Bornstein B, Broicher A, Courtot M, Donizelli M (2006). BioModels Database: a free, centralized database of curated, published, quantitative kinetic models of biochemical and cellular systems.. Nucleic Acid Research.

[22] Gilbert D, Heiner M (2006). From Petri nets to differential equations - an integrative approach for biochemical network analysis..

[23] Gend CV, Kummer U, Yi TM, Hucka M, Morohashi M, Kitano H (2001). STODE - automatic stochastic simulation of systems described by differential equations.. Proceedings of the 2nd International Conference on Systems Biology.

[24] Dematté L, Priami C, Romanel A, Bernardo M, Degano P, Zavattaro G (2008). The BlenX language: A tutorial.. 8th Int. School on Formal Methods for the Design of Computer, Communication and Software Systems: Computational Systems Biology SFM'08; volume 5016 of *Lecture Notes in Computer Science*.

[25] Dematte L, Priami C, Romanel A (2008). The beta workbench: a computational tool to study the dynamics of biological systems.. Brief Bioinform.

[26] Euler L (1809). De resolutione fractionum compositarum in simpliciores.. Mémoires de l′Académie des sciences de St-Pétersbourg.

[27] Bause F, Kemper P, Kritzinger P (1994). Abstract Petri Net Notation..

[28] Soliman S (2009). Modelling biochemical reaction networks with biocham extracting qualitative and quantitative information from the structure. In: Proceedings of the 6th Vienna Conference on Mathematical Modelling MATHMOD'09.. ARGESIM, volume.

[29] Yamada S, Shiono S, Joo A, Yoshimura A (2003). Control mechanism of jak/stat signal transduction pathway.. FEBS Letters.

[30] Singh A, Jayaraman A, Hahn J (2006). Modeling regulatory mechanisms in il-6 signal transduction in hepatocytes.. Biotechnology and Bioengineering.

[31] Rohr C, Marwan W, Heiner M (2010). Snoopy - a unifying Petri net framework to investigate biomolecular networks.. Bioinformatics.

[32] Funahashi A, Matsuoka Y, Jouraku A, Morohashi M, Kikuchi N (2008). Celldesigner 3.5: A versatile modeling tool for biochemical networks.. Proceedings of the IEEE.

[33] Brightman F, Fell D (2000). Differential feedback regulation of the MAPK cascade underlies the quantitative differences in EGF and NGF signalling in PC12 cells.. FEBS letters.

